# Effects of High-Velocity Elbow Manipulation on Forearm Muscle Electromyographic Recovery in Karting Drivers: A Randomized Within-Participant Sham-Controlled Trial

**DOI:** 10.3390/jcm15114267

**Published:** 2026-05-31

**Authors:** Rafał Studnicki, Aleksander Zarembski, Julia Wasilewska, Bartosz Trąbka

**Affiliations:** 1 Student Scientific Circle of Orthopaedic Manual Therapy, Medical University of Gdańsk, 7 Dębinki Street, 80-211 Gdańsk, Poland; aleksander.zarebski@gumed.edu.pl; 2 Department of Physiotherapy, Medical University of Gdańsk, 7 Dębinki Street, 80-211 Gdańsk, Poland; julia.wasilewska@gumed.edu.pl; 3 Fitness Department, University of Physical Education and Sport in Gdansk, Kazimierza Górskiego 1 Street, 80-336 Gdańsk, Poland; bartosz.trabka@gda.pl

**Keywords:** karting, elbow manipulation, forearm electromyography, neuromuscular activation, training load

## Abstract

**Objectives**: Karting imposes high neuromuscular demands on the forearm during dynamic steering, gripping and braking. This study examined whether a single high-velocity, low-amplitude (HVLA) manipulation of the elbow acutely modified surface EMG_RMS amplitude and EMG median frequency responses during standardized isometric forearm testing after simulated karting load, rather than EMG activity during dynamic driving itself. **Methods**: In this randomized, sham-controlled, within-subject trial, 15 drivers completed a single-session within-participant protocol in which one upper limb was randomly allocated to receive elbow HVLA manipulation (manipulated limb) and the contralateral limb received a standardized sham procedure (sham limb) involving therapist contact and low-grade oscillatory movement without end-range pre-tension or thrust. Drivers completed two 8 min simulated races separated by the allocated manual procedure. Surface electromyography (EMG) from four forearm muscles was collected outside the karting task during standardized laboratory-based isometric forearm contractions at baseline, after race 1, post-intervention, and after race 2. EMG was not recorded during real-time steering, braking, vibration exposure or competitive driving. The extensor carpi radialis (ECR) was specified as the principal muscle of interest because the HVLA technique pre-tensioned the common extensor origin and radial wrist extensors. The primary outcome was ECR mean EMG_RMS amplitude, expressed in µV, across the four measurement time points; the primary statistical test was the condition × time interaction. ECR maximal EMG_RMS amplitude and ECR median frequency were treated as secondary outcomes, whereas ECU, FCR, and FCU outcomes were treated as exploratory anatomical specificity outcomes. Mixed-model ANOVAs compared maximal and mean EMG amplitudes and median frequency between manipulated and sham limbs, treating limb condition and time as repeated within-participant factors. **Results**: For the primary outcome, ECR mean EMG_RMS amplitude showed a main effect of condition (*p* = 0.023) and a condition × time interaction (*p* < 0.001). As a secondary amplitude outcome, ECR maximal EMG_RMS amplitude showed a main effect of time (*p* = 0.009) and a condition × time interaction (*p* < 0.001), with higher post-manipulation values in the manipulated limb. No consistent limb-condition effects were found for the other muscles, and EMG median frequency showed only modest time-related changes (*p* = 0.031) without between-condition differences. **Conclusions**: A single-elbow manipulation produced short-lived, muscle-specific increases in ECR activation after simulated racing, whereas broader neuromuscular changes were not evident. These findings indicate only transient modulation of ECR surface EMG amplitude in a small sample of screened karting drivers and do not demonstrate improved recovery, neuromuscular efficiency, sport performance, or injury prevention. Because EMG was assessed during standardized isometric contractions rather than during dynamic steering, braking, vibration exposure or competitive racing, the findings should not be interpreted as direct evidence of altered neuromuscular behaviour during actual kart driving. Larger studies including force, performance, clinical, fatigue-specific and dynamic driving EMG outcomes are required.

## 1. Introduction

The elbow is a complex trochoginglymus joint composed of the humeroulnar, humeroradial and proximal radioulnar articulations, stabilized by capsuloligamentous structures and dynamically controlled by synergistic flexor–extensor and pronator–supinator muscle groups to position the hand and transmit forces between the upper limb and the external environment [1]. Elbow disorders relevant to sport and manual therapy should be distinguished clinically because they differ in mechanism, prognosis, contraindications and therapeutic implications. Because of its role in repetitive gripping and forearm rotation, the elbow is susceptible to overuse-related conditions, including lateral and medial elbow tendinopathy, when exposed to repetitive, forceful and awkward upper-limb tasks [2]. Population-based studies have reported point or lifetime prevalences of epicondylitis in the order of 1–3% and have identified repetitive wrist and forearm movements, high force, and vibration as key occupational risk factors, underscoring the substantial clinical and socioeconomic burden of elbow overuse disorders [3]. Lateral elbow tendinopathy is best understood as a load-related disorder of the common extensor origin, particularly involving the extensor carpi radialis brevis region, rather than as a purely inflammatory condition [4,5].

In athletes, especially those engaged in overhead throwing, racquet and stick sports, the elbow is subjected to extreme valgus and extension loads that produce a characteristic pattern of soft-tissue and osteochondral overuse injuries [6]. Lateral elbow tendinopathy in this context reflects a degenerative process of the common extensor origin driven by repetitive, high-load gripping and wrist extension rather than a purely inflammatory pathology, with pain and weakness often limiting performance [7]. This symptomatic tendinopathy construct should be distinguished from exercise-induced forearm fatigue in otherwise healthy athletes. In the latter context, repeated gripping, steering or braking may alter neuromuscular activation without necessarily indicating tendon pathology, joint stiffness or clinically diagnosed elbow disease. Beyond classic overhead and racquet sports, grip-intensive disciplines such as rowing, climbing and motorsport require sustained isometric contraction of the wrist and finger flexors, and recent work in high-level riders and drivers indicates that such tasks can provoke substantial forearm neuromuscular fatigue consistent with an overuse mechanism affecting the elbow–forearm complex [8,9]. Within this common-extensor complex, the extensor carpi radialis brevis region is frequently emphasized in lateral elbow pain and tendinopathy models, and repetitive loaded gripping and wrist extension are central provocative demands [4].

Contemporary motorsport has been shown to impose cardiovascular and neuromuscular demands on drivers that are comparable to those of other high-intensity sports, with elevated heart rate and oxygen consumption reflecting the combined effects of G-forces, heat stress and sustained muscle activation [10]. Field interviews and performance analyses in stock-car racing have characterized motorsport athletes as experiencing considerable physical strain and a notable incidence of musculoskeletal injuries, frequently involving the upper extremity, while often lacking structured conditioning programs tailored to these demands [11]. Case series and registry-based reports on go-kart accidents further demonstrate a substantial burden of injuries, including fractures and soft-tissue trauma of the upper limb, highlighting karting as a sport with non-trivial risk to the elbow and forearm despite its “recreational” image [12,13]. Acute traumatic injuries are clinically distinct from overuse-related forearm fatigue and lateral elbow tendinopathy. In particular, elbow fracture, dislocation, surgery, prolonged immobilization and periarticular soft-tissue injury may lead to post-traumatic elbow stiffness, a condition characterized by capsular contracture, heterotopic ossification, osseous impingement or combined structural restriction rather than by acute exercise-induced neuromuscular fatigue [14,15].

Post-traumatic elbow stiffness is relevant to karting because traumatic upper-limb injuries and fractures can occur in go-kart accidents, but its management should not be conflated with the intervention tested in the present study [12,13]. Recent evidence indicates that first-line management of post-traumatic elbow stiffness is generally conservative, commonly including physiotherapy, therapeutic exercise, splinting/bracing and non-thrust mobilization strategies. Surgical management, including open or arthroscopic arthrolysis, is usually reserved for complex cases with structural restriction or for patients who fail to achieve adequate improvement after conservative management [15,16,17,18,19]. Therefore, evidence supporting mobilization or rehabilitation for post-traumatic elbow stiffness does not automatically justify HVLA manipulation, particularly in the presence of recent fracture, instability, postoperative restriction, heterotopic ossification, or unresolved pain.

Electromyographic (EMG) studies in motorcycle riders show that repetitive braking and continuous gripping produce marked fatigue of the wrist and finger flexor muscles, implicating the same forearm musculature that contributes to steering and braking in karting and suggesting that these muscles may be particularly vulnerable to overload in motorsport settings [8,9]. The first EMG investigation specifically with regard to trained karting drivers demonstrated that even a short bout of driving (680 m) is sufficient to alter forearm muscle activation patterns and indicate emerging fatigue, supporting the view that karting imposes high neuromuscular demands on the elbow–forearm system [20]. The present trial was positioned within the neuromuscular load-and-recovery domain in healthy drivers, not within the treatment pathway for post-traumatic elbow stiffness or symptomatic lateral elbow tendinopathy.

Manual therapy is an umbrella term that includes several biomechanically distinct procedures, among which joint mobilization and high-velocity, low-amplitude (HVLA) manipulation should not be considered interchangeable. Joint mobilization generally refers to low-velocity, non-thrust, sustained or oscillatory passive movement applied within or near the available range of motion, whereas HVLA manipulation involves a rapid, low-amplitude thrust delivered at or near an end-range barrier [21]. These differences are relevant because force–time characteristics, dosage, sensory input, expected neurophysiological responses, indications and safety considerations may differ between mobilization and manipulation. Accordingly, evidence from mobilization studies can support the broader biological plausibility that articular manual input may influence pain, grip force or neuromuscular function, but it cannot be assumed to demonstrate the specific effects of elbow HVLA manipulation. Contemporary models propose that manual mechanical stimuli may initiate peripheral and central neurophysiological responses, but the magnitude and direction of these responses are likely to depend on the technique, target tissue, dosage, participant phenotype and outcome timing [22].

In patients with lateral epicondylalgia, non-thrust manual approaches such as mobilization-with-movement and thoracic spinal mobilization have been associated with immediate improvements in pain-free grip strength and related clinical outcomes, suggesting that articular manual input may acutely influence upper-limb function [23]. However, these studies should be interpreted as indirect support for the present trial because they evaluated mobilization-based procedures rather than elbow HVLA thrust manipulation. In contrast, neurophysiological studies of spinal HVLA manipulation provide more specific evidence that thrust manipulation can alter sensorimotor excitability and cortical drive to limb muscles, although these findings remain anatomically indirect because they were obtained from spinal rather than elbow interventions [24]. Surface electromyography (EMG) has been advocated as a useful method to assess short-term neuromuscular responses after manual interventions, but interpretation should distinguish whether the applied procedure is mobilization, manipulation, soft-tissue treatment or a multimodal intervention.

Despite these advances, most trials of manual procedures for lateral elbow pain have been conducted in clinical populations at rest and have often evaluated mobilization-based, multimodal, or non-thrust approaches, rather than isolating the acute effects of a standardized elbow HVLA manipulation under sport-specific loading conditions [25]. Likewise, motorsport EMG research in riders and drivers has focused on characterizing forearm muscle fatigue during riding or gripping tasks but has not examined whether a joint-specific HVLA thrust manipulation can acutely modulate these neuromuscular responses under sport-specific conditions [8,9]. To our knowledge, no previous study has evaluated whether a targeted elbow HVLA thrust technique applied to the radiohumeral joint region can acutely modify surface EMG_RMS amplitude or EMG median frequency responses of forearm muscles during standardized isometric testing performed after simulated karting load in trained drivers, representing a focused physiological gap given the documented forearm loading in this sport [20].

Therefore, the primary objective of the present study was to determine whether a standardized elbow HVLA manipulation, rather than manual therapy in general, acutely modified ECR mean EMG_RMS amplitude in amateur karting drivers after simulated karting load, using standardized isometric laboratory tests as the EMG assessment condition. Secondary objectives were to examine ECR maximal EMG_RMS amplitude and ECR median frequency, whereas EMG outcomes from ECU, FCR, and FCU were analysed exploratorily to assess whether any response was anatomically specific to ECR or reflected a broader forearm response. Handgrip force, elbow range of motion, upper-limb kinematics, neuromuscular efficiency, sport performance, pain, clinical recovery and injury-prevention outcomes were outside the analytical scope of this report. This design separated the dynamic load exposure from the EMG measurement task: the simulated races were used to impose karting-specific forearm load, whereas EMG was recorded during controlled isometric contractions to improve signal standardization, repeatability and between-condition comparability. Consequently, the study was not designed to characterize EMG activity during real-time steering, braking, vibration exposure, or psycho-emotional racing stress. Surface EMG amplitude is highly task- and context-dependent and should be interpreted within the specific recording and contraction conditions used [26].

Because evidence from spinal manipulation and thoracic mobilization cannot be assumed to generalize directly to peripheral elbow thrust manipulation, the present hypothesis was exploratory and based on biological plausibility rather than on established elbow-specific neurophysiological evidence. The clinical rationale was not that HVLA manipulation is a treatment for post-traumatic elbow stiffness or diagnosed lateral elbow tendinopathy; rather, it was that karting imposes high repetitive neuromuscular demands on the elbow–forearm complex and that a single peripheral thrust manipulation may plausibly alter short-term neuromuscular activation in a screened, non-injured athletic population. Based on evidence that thrust manipulation can acutely influence sensorimotor excitability and voluntary muscle activation, and on indirect evidence that non-thrust mobilization procedures may affect pain-free grip and upper-limb function [27,28], we hypothesized that, compared with the sham limb condition, the HVLA manipulative technique would acutely modify ECR mean EMG_RMS amplitude, with ECR maximal EMG_RMS amplitude and ECR median frequency analysed as secondary electrophysiological outcomes. We did not hypothesize effects on elbow range of motion, handgrip force, upper-limb kinematics, neuromuscular efficiency, sport performance, pain, clinical recovery or injury prevention in the present report, because these outcomes were not part of the current analysis. Because mobilization and manipulation differ biomechanically, the mobilization literature was used only to support biological plausibility and not as direct evidence of the efficacy of elbow HVLA manipulation. Moreover, this rationale concerns short-term neuromuscular modulation in a screened athletic population and should not be interpreted as evidence that elbow HVLA manipulation is safe or effective for pathological elbow conditions such as post-traumatic stiffness, recent fracture or dislocation, active lateral elbow tendinopathy, neuropathic elbow pain, inflammatory disease, instability, or undiagnosed elbow pain. HVLA techniques involve rapid low-amplitude thrust loading, and authoritative clinical descriptions emphasize that acute injury, undiagnosed disorders, altered joint morphology, recent surgery, instability, dislocation, bone disease, neurological disorders, and severe pain require caution or may contraindicate thrust manipulation. Thus, any observed EMG changes were therefore interpreted as short-term neuromuscular responses to the tested peripheral elbow procedure, not as proof that spinal and peripheral manipulations share identical mechanisms. Moreover, given the heterogeneity of elbow conditions and manual therapy techniques, this hypothesis was considered exploratory and was not intended to support HVLA manipulation in traumatic, postoperative, stiff or unstable elbows. Finally, because EMG amplitude alone cannot establish improved force production, recovery, neuromuscular efficiency, sport performance, or injury prevention, these outcomes were interpreted as exploratory physiological markers rather than direct indicators of clinical or performance benefit.

## 2. Materials and Methods

### 2.1. Study Design and Setting

This investigation used a single-, randomized, counterbalanced, within-participant split-body, sham-controlled trial design with repeated measures at four time points to evaluate the effect of elbow joint manipulation on forearm muscle electromyographic recovery in adult go-kart drivers. Each participant contributed paired data from two upper limbs: one limb randomized to HVLA manipulation and the contralateral limb assigned to the sham comparator. Therefore, the participant, not the elbow, was the unit of statistical independence, and limb-level observations were treated as correlated repeated measurements nested within participant. The trial is reported in accordance with the CONSORT 2025 statement [29], with additional consideration of the CONSORT extension [30] for within-person randomized trials because the intervention contrast was made between paired limbs within the same participant. Thus, the trial was not a parallel-group randomized trial and did not include an untreated contralateral comparator. Instead, each participant contributed paired limb data: one upper limb was randomly allocated to receive the high-velocity, low-amplitude (HVLA) elbow manipulation, whereas the contralateral limb received a standardized sham procedure.

**Figure 1:**
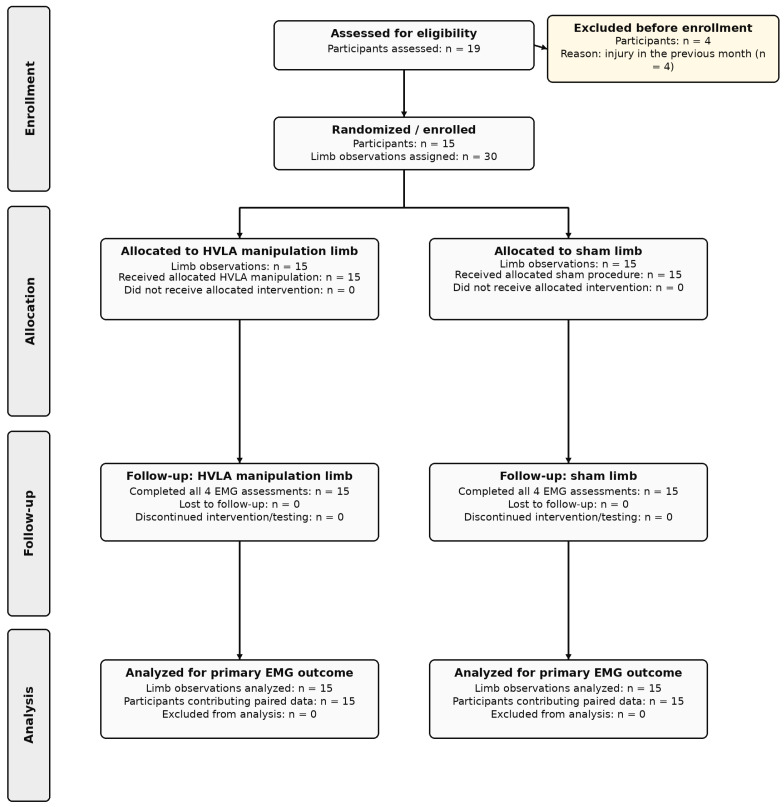
Participant flow diagram for the within-participant limb-condition trial. HVLA: high-velocity, low-amplitude. Limb conditions were classified as manipulated limb and sham limb.

For each participant, one upper limb was randomly allocated to receive the high-velocity, low-amplitude (HVLA) elbow manipulation (manipulated limb), whereas the contralateral limb received a standardized sham manual procedure (sham limb). The comparator was therefore not an untreated or non-manipulated control limb. It was a sham limb designed to approximate the contextual and procedural features of the active intervention while omitting end-range pre-tension and the rapid HVLA thrust. The allocation of dominant versus non-dominant limb to the manipulated condition was counterbalanced across drivers to avoid systematic confounding by limb dominance. For consistency, the two limb conditions are referred to throughout the manuscript as the “manipulated limb” and the “sham limb.” This terminology was used because the sham limb received therapist contact, limb positioning, joint handling, and low-grade non-thrust sham movement, and therefore should not be described as untreated or merely non-manipulated. Because dominance and anatomical side may still influence forearm EMG amplitude and fatigue responses, dominance/laterality was also examined in exploratory sensitivity analyses. The study was prospectively registered at ClinicalTrials.gov (NCT06826300) and approved by the Independent Bioethics Committee for Scientific Research at the Medical University of Gdańsk (Resolution No. KB/519/2024). The trial was conducted in accordance with the ethical principles of the Declaration of Helsinki, and all participants provided written informed consent after receiving verbal and written information about the aims, procedures, potential risks and their right to withdraw at any time without consequences. The participant flow is shown in Figure 1.

No changes were made to eligibility criteria, the intervention or sham procedures, or the four EMG measurement time points after trial commencement. For inferential hierarchy, ECR mean EMG_RMS amplitude was defined as the single primary outcome; ECR maximal EMG_RMS amplitude and ECR median frequency were secondary outcomes, and all non-ECR forearm EMG outcomes were exploratory.

All EMG assessments were performed in the same dedicated examination room, using the same treadmill and sEMG system, under controlled environmental conditions of 23 °C and 55% relative humidity, with only one participant present at a time to minimize discomfort and distraction. Two investigators were involved throughout, a physiotherapist with expertise in manual therapy, responsible for delivering the elbow manipulation or sham procedure, and an examiner experienced in EMG who was responsible for electrode preparation, signal acquisition and data quality control. Karting-specific effort was standardized by having participants complete driving bouts that replicated their usual training intensity on the same go-kart circuit, using 8 min race simulations as the standardized karting-specific load exposure. The protocol deliberately separated the load-exposure condition from the EMG measurement condition. The simulated races served only to impose a karting-related forearm load, including sustained gripping and steering demands. In contrast, the EMG outcomes reported in this manuscript were obtained before and after these load exposures during standardized laboratory-based isometric forearm contractions, with the forearm supported, joint position controlled, and resistance standardized. No EMG signal was recorded continuously during the simulated races, real-time steering, braking, vibration exposure or race-like psychological stress. Therefore, all reported EMG_RMS amplitudes and median frequency outcomes should be interpreted as post-load laboratory isometric EMG responses, not as direct measures of in-kart muscle activation. This approach was chosen to reduce movement artefact, standardize joint position and resistance, and permit paired within-participant comparison between manipulated and sham limbs, but it necessarily reduced ecological validity relative to in-kart EMG recording.

Each eligible driver attended a single study session, during which all measurements and interventions were completed. After arrival and confirmation of inclusion and exclusion criteria (licensed karting driver actively training in a league, age ≥ 18 years, good general health, no upper-extremity injury in the previous six months, and no relevant neurological or connective tissue disease), participants completed a standardized warm-up consisting of 10 min of dynamic upper-limb stretching followed by 5 min of isometric upper-limb exercises. Baseline surface EMG recordings (Measurement 1) were then obtained from the selected forearm muscles, after which participants undertook the first 8 min race. Immediately after this race, a second EMG assessment (Measurement 2) was performed to characterize the acute neuromuscular response to driving. Participants then received both allocated limb procedures within the same session: the limb randomized to the active condition received the high-velocity, low-amplitude lateral elbow thrust, whereas the contralateral limb received the standardized sham procedure. A third EMG assessment (Measurement 3) was performed immediately post-intervention to capture the short-term laboratory isometric EMG response to manipulation versus sham, followed by a second 8 min race and a final EMG assessment (Measurement 4), again performed outside the karting task during standardized isometric contractions, to evaluate post-load EMG response patterns across consecutive simulated race exposures. Electromyographic activity was recorded bilaterally at each time point so that neuromuscular responses could be compared within participants between the manipulated limb and the sham limb across the four measurement occasions.

### 2.2. Participants

Participants were recruited using a non-probability, consecutive sampling strategy from licensed go-kart drivers actively training and competing in karting leagues who were invited to take part in the study during routine training sessions and league events. Drivers were informed about the study in person by the investigators and via announcements distributed through collaborating karting clubs, and those who expressed interest were contacted for eligibility screening. All screening and enrolment procedures were conducted at the university-affiliated sports science and rehabilitation facility where the experimental sessions took place.

Inclusion and exclusion criteria were defined a priori to obtain a homogeneous sample of healthy, actively competing karting drivers and to minimize confounding by pre-existing musculoskeletal or systemic conditions. To be included, participants had to (i) be actively training as a licensed driver competing in a karting league; (ii) be in good general health with no recent history of injury or acute illness; (iii) be at least 18 years of age; and (iv) be able and willing to complete all phases of the experimental protocol, including both simulated races and all electromyographic assessments. Exclusion criteria were (i) sustaining an injury or illness during the study period that could interfere with driving or EMG measurements; (ii) a history of upper-extremity injury (shoulder, elbow or wrist), pain in any of these joints, or upper-extremity surgery within the previous six months; (iii) clinically assessed hypermobility of a lower-extremity joint; or (iv) any diagnosed neurological or connective tissue disease. Drivers using medications or treatments that could plausibly affect neuromuscular function (e.g., recent corticosteroid injections or ongoing structured elbow rehabilitation) were also excluded or rescheduled until such interventions had been completed, to reduce potential bias in EMG outcomes. During screening, the investigator recorded past injuries, current symptoms, medical diagnoses and participation level in karting to confirm that all drivers met the criteria for being healthy, actively competing athletes. Candidates who did not satisfy all inclusion criteria or met any of the exclusion criteria were not enrolled and were informed about the reasons for their non-eligibility. For safety and external validity reasons, the target population was restricted to apparently healthy drivers. Drivers were not eligible if screening identified current elbow pain, recent elbow or upper-limb trauma, previous fracture or dislocation with residual symptoms, clinically apparent elbow stiffness or restricted range of motion, suspected instability, neurological symptoms such as paresthesia or radiating pain, active inflammatory or connective-tissue disease, current elbow rehabilitation, recent injection therapy, or postoperative restriction. These exclusions were applied because pathological elbow conditions may involve structural, osseous, capsular, tendinous, inflammatory, or neural mechanisms that require diagnosis-specific management and may alter the risk–benefit profile of HVLA manipulation [4,31].

A total of 15 licensed karting drivers met the eligibility criteria, provided informed consent and completed all four EMG assessments and both 8 min race simulations, resulting in a complete dataset with no loss to follow-up or missing outcome measurements for the primary EMG outcome, ECR mean EMG_RMS amplitude, or for the secondary and exploratory EMG variables. The sample consisted of 15 male amateur, though competitively active, drivers regularly training in karting leagues; all were adults aged 18 years or older. The mean age was 23.5 ± 3.1 years. All participants were licensed karting drivers actively training and competing in organized karting leagues, corresponding to an amateur competitive level rather than professional motorsport participation. Anthropometric data obtained at baseline showed a mean body mass of 77.9 ± 13.5 kg (range 52–98 kg) and a mean body height of 179.5 ± 9.1 cm (range 158–193 cm), corresponding to a mean body mass index of 24.0 ± 2.9 kg·m^−2^ (range 19.5–29.3 kg·m^−2^). Drivers had 3.2 ± 0.9 years of karting experience and trained 3.1 ± 1.3 sessions per week.

### 2.3. Limb-Level Intervention Conditions

#### 2.3.1. Manipulated Limb

For each participant, one elbow was randomly allocated to the manipulated-limb condition. The participant lay supine on a plinth with the shoulder in slight abduction and neutral rotation, the elbow of the manipulated limb close to full extension, and the forearm supported but free to move in pronation–supination.

The limb allocated to the manipulated condition received a single standardized high-velocity, low-amplitude (HVLA) manipulation directed to the elbow joint instrumented with surface EMG electrodes. The participant lay supine on a plinth with the shoulder in slight abduction and neutral rotation, the elbow close to full extension, and the forearm supported but free to move in pronation–supination. The therapist stood on the lateral side of the tested limb and established firm contact with the medial aspect of the distal humerus just proximal to the elbow joint line using the hypothenar eminence of the cranial hand, while the caudal hand grasped the participant’s distal forearm and wrist.

To pre-tension the common extensor origin and radial wrist extensors, the forearm was positioned in maximal comfortable pronation, the wrist in flexion combined with slight ulnar deviation, and the elbow taken to the end of its available extension range until a firm capsular end-feel was perceived. This pre-position is consistent with classical descriptions of Mill’s-type elbow manipulation, in which an HVLA thrust into the end range of elbow extension is applied with the wrist flexed and the forearm pronated to tension the teno-osseous junction at the lateral epicondyle [4,32]. From this pre-loaded position, the therapist applied a progressive lateral distraction–glide to the ulnohumeral/radiocapitellar complex by translating the distal humerus medially relative to the ulna and radius (or conversely the forearm laterally relative to the humerus), increasing tissue tension through oscillatory movements corresponding to Maitland grades II–III while continuously monitoring patient comfort.

Once a clear end-range resistance barrier was appreciated at grade III, the therapist maintained this position for approximately 2 s and then delivered a single, quick HVLA lateral thrust of small amplitude directed perpendicular to the long axis of the humerus, aiming to gap the lateral compartment of the elbow without forcing further extension. This thrust magnitude and direction were chosen to respect the physiological range of the joint and to be consistent with published descriptions of elbow HVLA techniques used in the management of lateral elbow tendinopathy [32]. The manipulation was performed once per experimental session on the tested elbow, with no repeated thrusts unless the initial thrust was clearly incomplete (e.g., loss of contact or unexpected participant movement); in such cases, a maximum of one additional thrust was allowed after re-establishing the pre-load position. These oscillatory movements were used only to establish tissue preload and participant comfort before the thrust; they were not intended to constitute a therapeutic mobilization dose. The active intervention was defined a priori as the subsequent single HVLA thrust.

Throughout the procedure, participants were instructed to remain relaxed and to report any pain or discomfort; the technique was immediately aborted if sharp pain, paresthesia or a sense of instability occurred. The HVLA thrust was applied only after eligibility screening excluded recent trauma, current pain, neurological symptoms, instability, postoperative restriction, and clinically apparent elbow stiffness. The procedure was not intended for drivers with suspected fracture, dislocation, post-traumatic stiffness, active tendinopathy, neuropathic pain, inflammatory arthropathy, osseous deformity, heterotopic ossification, or undiagnosed elbow symptoms. In such conditions, thrust manipulation may be inappropriate or require prior medical evaluation, imaging, or condition-specific rehabilitation [17]. No local anaesthetic, analgesic premedication or adjunct physical agents (e.g., ultrasound, bracing or taping) were used, in order to isolate the effect of the manipulative thrust. The entire manipulation sequence from initial positioning to completion of the thrust lasted approximately 30–60 s. Immediately after the intervention, participants remained on the plinth for 1–2 min to ensure that they experienced no adverse effects such as dizziness or unexpected pain, after which the next EMG assessment (Measurement 3) was performed.

The selection of a single-session elbow HVLA manipulation was based primarily on its clinical use in lateral elbow conditions and on the need to test an isolated peripheral thrust procedure under sport-specific conditions. Evidence from thoracic mobilization and spinal HVLA manipulation was considered mechanistically informative but indirect, because spinal and peripheral joint procedures differ in anatomical target, afferent input, force application, expected neurophysiological response, and clinical indication [27,33]. Accordingly, the present protocol was designed to evaluate the immediate EMG response to elbow HVLA manipulation itself rather than to infer its effects from spinal manual therapy literature.

#### 2.3.2. Sham Limb Comparator

The contralateral elbow of each participant served as the sham limb comparator and received a standardized sham manual procedure during the same experimental session. This limb was instrumented with sEMG electrodes in the same manner as the manipulated limb and was subjected to the same warm-up, simulated driving bouts, and EMG assessment procedures at all four time points. The sham procedure was not an untreated control condition. Rather, it was designed to preserve procedural identity with the active intervention as far as feasible in a manual therapy trial by reproducing therapist presence, manual contact, participant positioning, joint handling, and approximate duration while omitting the hypothesized active components of the HVLA intervention: end-range pre-tension and the rapid thrust [34]. This design allowed within-participant comparison of neuromuscular responses between the manipulated limb and sham limb while holding constant participant-level characteristics, bilateral EMG instrumentation, simulated driving exposure, therapist contact, limb handling, approximate procedure duration, and assessment timing.

The therapist placed the hands in the same positions as for the active manipulation (cranial hand on the medial distal humerus, caudal hand on the distal forearm), and brought the elbow into a near-extension position within the mid-range of motion, but deliberately stopped short of the true end range used in the HVLA technique. The forearm was placed in neutral to mild pronation, and the wrist was held in a neutral position without the combined flexion and ulnar deviation used to pre-tension the common extensor origin in the manipulated-limb condition. A gentle lateral–medial oscillatory movement corresponding to low-grade, non-thrust sham movement was then applied for approximately 20–30 s to simulate manual contact and movement, without delivering a therapeutic mobilization dose, end-range pre-tension, or HVLA thrust. However, no rapid thrust, no sudden increase in force, and no further excursion into end-range extension were performed.

Crucially, the sham procedure reproduced the visual and tactile experience of the active intervention (same therapist, body contact, joint handling, and approximate time duration) while omitting the key elements that distinguished the experimental procedure as HVLA manipulation, namely end-range pre-tension and the rapid low-amplitude thrust. Thus, the comparator should be interpreted as a sham manual-contact condition rather than as an active joint mobilization intervention [34]. Similar sham designs, in which joint positioning and light manual contact are maintained but the thrust component is removed, have been successfully used in randomized trials of cervical and thoracic spine manipulation and in studies of elbow mobilization, demonstrating that such procedures can maintain participant blinding and serve as credible placebos [35,36]. Participants were informed that each elbow would receive a different manual procedure used in physiotherapy practice, but they were not told which procedure was hypothesized to be active.

The sham procedure was designed to approximate the contextual features of the active procedure, including therapist presence, manual contact, limb handling, participant positioning and approximate duration, while omitting end-range pre-tension and the HVLA thrust. However, sham credibility was not formally tested in this study. Participants’ treatment expectations, prior experience with manual therapy or manipulation, perceived limb allocation, and confidence in the credibility of each procedure were not assessed with validated questionnaires. Consequently, the sham condition should be interpreted as a procedural control for therapist contact and handling rather than as a fully validated placebo control.

### 2.4. Evaluation Procedures

All outcome assessments were carried out in a dedicated room using the same equipment for all participants to ensure consistency and reduce measurement bias. The room temperature was maintained at 23 °C with 55% relative humidity, and each participant was assessed individually to minimise external distractions and discomfort. The evaluation protocol comprised four surface electromyography (sEMG) measurement time points: baseline before any simulated race (Measurement 1), immediately after the first 8 min race (Measurement 2), immediately after the allocated intervention (elbow manipulation or sham; Measurement 3), and immediately after the second 8 min race (Measurement 4). These four EMG assessments were not in-kart recordings. Each assessment was performed under standardized laboratory conditions during controlled isometric forearm contractions. Thus, the simulated races represented the dynamic load stimulus, whereas the EMG assessments represented controlled post-load measurement epochs. At each time point, sEMG recordings were obtained from pre-specified forearm muscles on both upper limbs using identical electrode placements and acquisition parameters. Before the first EMG assessment, participants completed a standardised warm-up consisting of 10 min of dynamic upper-limb stretching and 5 min of isometric upper-limb exercises led by the investigator, after which they were familiarised with the EMG equipment and testing procedures. For analysis, these recordings were classified according to randomized limb allocation (manipulated limb vs. sham limb), enabling within-participant comparison of neuromuscular responses between the elbow that received HVLA manipulation and the contralateral elbow that received the standardized sham procedure across all four measurement times.

Surface EMG recordings were used as the principal method to quantify neuromuscular activation and fatigue of the forearm musculature, given the established validity of sEMG for assessing muscle activation patterns and fatigue-related changes in amplitude and spectral content in both laboratory and sport-specific tasks [37]. All EMG signals were acquired using a TeleMyo DTS wireless system (Noraxon, Scottsdale, AZ, USA), a research-grade multichannel sEMG device that provides differential amplification and direct digital transmission of signals from the sensor to the receiver, thereby reducing cable artefacts and enhancing signal quality. Single-use Ag/AgCl surface electrodes with a circular recording area of 1 cm^2^ (Sorimex, Toruń, Poland) were applied in bipolar configuration with an inter-electrode distance of approximately 20 mm, in line with European SENIAM recommendations on electrode size and inter-electrode spacing for kinesiological EMG [38].

Signals were differentially amplified with a gain of 500, band-pass filtered between 15 and 500 Hz, and sampled at 1500 Hz with 16-bit resolution using an analogue-to-digital converter integrated with the Noraxon system. These acquisition parameters are consistent with contemporary methodological guidance for EMG analysis of upper-limb muscles during dynamic and fatiguing tasks [39]. Raw data were stored and processed offline using MyoResearch 2.8 software (Noraxon), which allowed uniform application of filtering, rectification and feature extraction procedures across all participants and sessions. Before each recording block, all channels were checked with the participant relaxed to verify stable baseline activity, absence of visible motion artefact, and adequate signal-to-noise ratio. The system was prepared and checked according to the manufacturer’s acquisition procedures, and electrode–skin contact was optimized through shaving, light abrasion, alcohol cleaning, secure fixation, and real-time inspection of each channel. If excessive baseline noise, poor signal discrimination, or unstable contact was observed, the electrode placement was adjusted before data acquisition. No additional hardware calibration factor was applied because the Noraxon system directly exports EMG amplitude in microvolts after analogue-to-digital conversion using the fixed acquisition settings described above.

Electrode placement and skin preparation adhered strictly to the surface EMG for non-invasive assessment of muscles (SENIAM) recommendations, which provide standardised procedures for electrode design, placement and signal processing to improve reliability and comparability between EMG studies [38]. Before electrode application, the skin over each recording site was shaved if necessary, lightly abraded and cleaned with 70% alcohol to reduce impedance and improve signal-to-noise ratio, as recommended in methodological and forearm-specific EMG studies [40]. Electrodes were placed parallel to the presumed orientation of muscle fibres and firmly secured with hypoallergenic tape to minimise movement artefact and cable-related noise. sEMG activity was recorded bilaterally from four forearm muscles: the extensor carpi radialis (ECR), the extensor carpi ulnaris (ECU), the flexor carpi radialis (FCR), and the flexor carpi ulnaris (FCU), which are the main contributors to wrist extension/flexion and ulnar/radial deviation during gripping and steering tasks in karting. ECR was selected as the principal muscle of interest because it represents the radial wrist extensor compartment targeted by the lateral elbow HVLA pre-position and contributes to wrist extension/radial-deviation control during gripping. ECU, FCR, and FCU were included to test whether any response was anatomically specific to ECR or reflected a more generalized forearm response.

Based on previous anatomical descriptions and EMG placement work [41,42], the ECR electrodes were positioned over the most prominent portion of the muscle belly approximately 3 cm distal to the lateral epicondyle along the line towards the radial styloid; ECU electrodes were placed at the midpoint between the lateral epicondyle and the ulnar styloid process on the dorsal–ulnar aspect of the forearm; FCR electrodes were located in the proximal third of the line between the medial epicondyle and the FCR tendon at the wrist on the volar–radial aspect; and FCU electrodes were placed over the muscle belly on the volar–ulnar side at approximately 70–80% of the distance between the pisiform and the medial epicondyle, as these locations have been shown to yield reliable and high-quality sEMG recordings from the respective muscles. A reference (ground) electrode was placed over an electrically neutral bony prominence (such as the ulnar styloid or olecranon), away from major muscle bellies.

During the initial assessment, participants were introduced to the EMG procedures and performed a “targeting phase” consisting of three brief, isolated voluntary contractions for each of the four forearm muscles to verify electrode placement, ensure clear signal discrimination and familiarise drivers with the required activation patterns; channels were visually inspected in real time and adjusted if necessary to optimise signal quality. This targeting phase was used for signal verification and task familiarisation only and was not used as a maximum voluntary contraction normalization trial. Assessment of muscle fatigue and the effect of manipulation on fatigue status was performed on both limbs across the four time points described above, with the same contraction tasks and instructions repeated to permit within-subject comparison over time. For each time point, sEMG recordings were obtained during standardised isometric contractions specific to the functional role of each muscle (wrist extension with ulnar/radial deviation for ECR and ECU and wrist flexion with ulnar/radial deviation for FCR and FCU), with the forearm supported and the joint angle and resistance standardised across participants. These tests were intended to provide a controlled physiological readout of forearm muscle excitation after the simulated driving load, not to reproduce the full dynamic neuromuscular demands of kart steering, braking or vibration exposure. Accordingly, the EMG outcomes should be interpreted as laboratory isometric responses measured after dynamic load exposure [37]. For quality control and potential secondary analyses of proximal muscle activity, additional sEMG recordings were obtained from the middle deltoid; posterior deltoid; upper, middle and lower trapezius; and infraspinatus during 3 s maximum voluntary isometric contractions (MVICs), using standard manual muscle testing positions and the same acquisition settings as for the forearm muscles. These proximal MVIC recordings were not used to normalize the primary forearm EMG outcomes. No forearm-specific MVC or MVIC normalization procedure was performed. Therefore, the primary ECR mean EMG_RMS outcome and the secondary/exploratory EMG amplitude outcomes are reported as absolute EMG_RMS amplitudes in µV rather than as percentage of MVC.

After band-pass filtering, all EMG signals were full-wave rectified and smoothed using the root mean square (EMG_RMS_) method with a 300 ms moving time window, which has been shown to provide a sensitive and reliable estimate of EMG amplitude, relatively robust to phase cancellation and suitable for quantifying changes in muscle activation during dynamic and fatiguing tasks [43]. For each muscle and time point, mean and maximal EMG_RMS amplitude (µV) were extracted over the contraction epochs of interest, computed separately for each trial and then averaged across trials. Only ECR mean EMG_RMS amplitude was treated as the primary outcome. ECR maximal EMG_RMS amplitude was treated as a secondary amplitude outcome, whereas mean and maximal EMG_RMS amplitudes from ECU, FCR, and FCU were treated as exploratory outcomes. Handgrip force, elbow range of motion, upper-limb kinematics, pain, clinical recovery, sport performance and injury-prevention endpoints were not collected or analysed in the present report.

Because the primary forearm EMG signals were not normalized to MVC/MVIC, these amplitude outcomes are reported as absolute microvolt values (µVs). Consequently, comparisons were interpreted primarily within the same muscle and within the same participant across time and limb condition, rather than as direct comparisons of activation magnitude between different muscles or between participants. The term “maximal EMGRMS amplitude” refers to the highest RMS-derived surface EMG amplitude observed within the analysed contraction epoch and should not be interpreted as mechanical force. EMG amplitude is an electrical measure of muscle excitation and cannot be equated directly with force production without simultaneous force measurement and appropriate modelling [26,44]. In the frequency domain, the median frequency of the power spectrum (EMG_MED_, Hz) of the raw sEMG signal was calculated using fast Fourier transform over the same contraction windows, as this parameter is sensitive to fatigue-related changes in muscle fibre conduction velocity and motor unit recruitment and shows acceptable reliability in dynamic and static tasks [45].

The decision to analyse absolute EMG_RMS amplitude was based on the same-session repeated-measures design: electrodes remained in place across the four assessments, testing positions and contraction instructions were standardized, and manipulated and sham limbs were compared within the same participant. This design reduces several sources of between-participant variability. However, because EMG amplitude normalization can materially affect interpretation, the absence of forearm MVC normalization is acknowledged as a limitation.

### 2.5. Randomization and Blinding

Within each participant, the side allocated to the manipulation condition (dominant vs. non-dominant upper limb) was randomized in a 1:1 ratio using a computer-generated allocation sequence created before the start of recruitment by an independent researcher not involved in enrolment, intervention delivery or outcome assessment. The sequence was produced with a random number generator using permuted blocks of variable size to maintain approximate balance in the number of dominant and non-dominant limbs assigned to the manipulation condition while preserving allocation unpredictability.

Allocation concealment was ensured through the use of sequentially numbered, opaque, sealed envelopes prepared by an administrative assistant who was not otherwise involved in the trial. For each enrolled driver, the envelope was opened only after eligibility had been confirmed, informed consent obtained, and baseline procedures (warm-up, Measurement 1 and the first 8 min race plus Measurement 2) completed. The envelope specified which upper limb (right vs. left; dominant vs. non-dominant) should receive the elbow manipulation, with the contralateral limb automatically assigned to the sham comparator condition.

The treating physiotherapist implemented the allocated intervention on the specified elbow, whereas the EMG examiner, who was responsible for electrode placement, signal acquisition and data quality control, remained outside the treatment area during the manipulation and was blinded to which limb had been manipulated during all subsequent EMG assessments. All EMG files and statistical datasets used coded variables (e.g., “Limb 1” and “Limb 2”) indicating manipulated versus sham limb condition without revealing side dominance, so that primary data processing and analyses could be conducted by a blinded analyst.

Because of the nature of the manual intervention, it was not possible to blind the treating physiotherapist to limb allocation, and participants may have perceived procedural differences between the thrust and sham procedures. However, both elbows received therapist contact and manual handling, and participants were not told which procedure was hypothesized to be active. Blinding success was not formally assessed and is acknowledged as a limitation. Participants were informed that both elbows would receive manual procedures commonly used by physiotherapists for elbow function, but they were not told which procedure was hypothesized to be active. Participant blinding was supported by applying therapist contact and joint handling to both limbs, with the sham procedure omitting only the end-range pre-tension and rapid thrust. Blinding success was not formally assessed, and this is acknowledged as a limitation. The EMG examiner, who was responsible for electrode placement, signal acquisition and data quality control, remained outside the treatment area during the intervention and was blinded to limb allocation during subsequent EMG assessments. All EMG files and statistical datasets used coded variables indicating manipulated versus sham limbs without revealing side dominance, so that primary data processing and analyses could be conducted by a blinded analyst. This approach ensured blinding of the outcome assessor and data analyst and minimized selection and detection bias within the constraints inherent to manual therapy research.

### 2.6. Sample Size

The a priori sample size was calculated for the single primary outcome, ECR mean EMG_RMS amplitude, comparing the manipulated and sham limb conditions across the four repeated measurement time points. The primary statistical test was the within-participant condition × time interaction for ECR mean EMG_RMS amplitude. ECR was selected as the target muscle because the HVLA pre-position loaded the common extensor origin and radial wrist extensor compartment, whereas the other forearm muscles were analysed as exploratory anatomical specificity outcomes. Based on previous EMG studies in karting drivers, where short driving bouts in 11–15 trained drivers produced clear within-subject changes in forearm muscle activation consistent with moderate-to-large effects on EMG amplitude, and on manual therapy trials in lateral epicondylalgia and spinal manipulation that typically report small-to-large effects on neuromuscular and functional outcomes, we anticipated at least a moderate condition × time effect for EMGRMS changes [20,33,46].

Reliability studies of upper-limb and forearm sEMG amplitude measures report good to excellent test–retest intraclass correlation coefficients (ICC = 0.70–0.95), supporting the use of repeated-measures designs with moderate correlations among time points [47,48]. Using G*Power 3.1, an a priori power analysis for a mixed-model repeated-measures ANOVA (within-participant condition × time interaction, two limb conditions, four measurement times, effect size f = 0.25 corresponding approximately to a moderate standardized difference, α = 0.05, power = 0.80, correlation among repeated measures set at 0.50 and nonsphericity correction ε = 1.0) indicated that a minimum of 24 participants would be required to detect the hypothesised condition × time interaction in EMG_RMS. The present trial enrolled and analysed 15 participants, each contributing paired data from one manipulated limb and one sham limb. Although this yielded 30 limb observations, these observations were not treated as statistically independent because limbs were clustered within participants. Consequently, the achieved sample did not meet the a priori participant-level target, and the study should be considered exploratory, with limited power to detect smaller condition × time effects or effects in muscles other than ECR.

### 2.7. Statistical Procedures

Before inferential analyses, data were screened for outliers, missing values and data-entry errors; distributional assumptions were assessed using Shapiro–Wilk tests, Q–Q plots, histograms and inspection of skewness and kurtosis for each outcome and time point. Because all enrolled participants completed the full protocol and none were lost to follow-up, the analyses followed a complete-case, intention-to-treat principle based on randomized limb-condition allocation.

The primary inferential analysis examined the effects of limb condition and time on ECR mean EMG_RMS amplitude, expressed in µV. Condition was treated as a within-participant factor with two levels: manipulated limb and sham limb. Time was treated as a within-participant repeated factor with four levels: pre-race baseline, post-race 1, post-intervention and post-race 2. Thus, the primary analysis explicitly accounted for the paired nature of the limb comparison within each participant. The primary statistical test was the condition × time interaction for ECR mean EMG_RMS amplitude.

Secondary analyses used the same modelling structure for ECR maximal EMG_RMS amplitude and ECR median frequency, because these variables provide complementary amplitude and spectral information from the target muscle. Exploratory analyses examined maximal EMG_RMS amplitude, mean EMG_RMS amplitude and median frequency for ECU, FCR, and FCU, as well as extensor–flexor EMG_RMS ratios, dominant–non-dominant limb differences, proximal shoulder–scapular EMG variables and laterality sensitivity analyses. These exploratory outcomes were used to assess anatomical specificity, possible broader forearm responses and robustness of the ECR findings, but they were not treated as additional primary outcomes.

Short-term neuromuscular effects of the intervention were summarized using the change in EMG outcomes between Measurement 2 (post-race 1) and Measurement 3 (post-intervention). These T2-to-T3 difference-in-differences analyses were treated as exploratory supportive analyses rather than as the primary statistical test. In parallel, linear mixed-effects models with random intercepts for participants and fixed effects for condition, time, and their interaction were fitted to confirm the robustness of the primary ECR mean EMG_RMS analysis and to account explicitly for within-subject correlation between limbs and across time. Holm–Bonferroni correction was applied to secondary and exploratory post-hoc comparison families; however, the inferential hierarchy was anchored on the single primary outcome, ECR mean EMG_RMS amplitude. To control the familywise error rate for multiple secondary EMG comparisons, Holm–Bonferroni correction was applied across the full family of limb-condition post-hoc tests, defined as 4 muscles × 3 EMG outcomes × 4 measurement time points, yielding 48 post-hoc comparisons. Both unadjusted and Holm-adjusted *p* values were inspected, but statistical significance for post-hoc tests was based on Holm-adjusted *p* values. Exploratory T2-to-T3 change-score comparisons were treated as a separate family of 12 tests, corresponding to 4 muscles × 3 EMG outcomes, and were also adjusted using Holm–Bonferroni correction. Effect sizes were expressed as partial eta-squared (ηp^2^) for ANOVA and as Cohen’s d for pairwise comparisons, each with 95% confidence intervals. For the key condition × time effects, 95% confidence intervals were also reported for partial eta-squared using the noncentral F distribution. To provide an interpretable estimate on the original EMG scale, the immediate post-procedure interaction was additionally summarized as a within-participant difference-in-differences contrast from post-race 1 to post-intervention: Δmanipulated limb − Δsham limb, with 95% confidence intervals expressed in µV. All analyses were conducted using standard statistical software (SPSS, version 28) for a *p* < 0.05.

As a robustness check, linear mixed-effects models were fitted with the participant as a random intercept and condition, time, and condition × time as fixed effects. These models were used to verify that the main conclusions did not depend on treating the paired limb measurements as independent observations. Pairwise post hoc comparisons were interpreted descriptively and adjusted using the Holm–Bonferroni method when omnibus tests were significant. No imputation was required because there were no missing primary EMG outcome data.

## 3. Results

Table 1 presents descriptive statistics for the primary, secondary and exploratory EMG outcomes in both limb conditions across the four assessment time points. The single primary outcome was ECR mean EMG_RMS amplitude, and the primary inferential test was the condition × time interaction across the four measurement time points. Post-hoc limb condition comparisons at individual time points were considered supportive and were not interpreted as independent primary tests. Holm–Bonferroni correction was applied across the pre-specified family of secondary and exploratory post-hoc comparisons.

After correction, the only between-condition differences that remained statistically significant were observed immediately after the allocated procedure for ECR maximal EMG_RMS amplitude and ECR mean EMGRMS amplitude. For ECR maximal EMG_RMS amplitude, the unadjusted post-race-1 difference between sham and manipulated limbs was no longer significant after correction (unadjusted *p* = 0.0079; Holm-adjusted *p* = 0.354). In contrast, the post-procedure difference remained significant after correction, with higher maximal EMG_RMS amplitude in the manipulated limb than in the sham limb (unadjusted *p* = 0.00022; Holm-adjusted *p* = 0.010). For ECR mean EMG_RMS amplitude, the unadjusted post-race-1 difference was also no longer significant after correction (unadjusted *p* = 0.0053; Holm-adjusted *p* = 0.243), whereas the post-procedure difference remained significant (unadjusted *p* = 0.00009; Holm-adjusted *p* = 0.004). No post-hoc between-condition comparisons for ECU, FCR, FCU, or median frequency outcomes remained significant after Holm–Bonferroni correction.

To quantify the key immediate interaction on the original EMG scale, we calculated the change from post-race 1 to post-intervention (T3–T2) for each limb and compared the within-participant between-condition change score, defined as Δmanipulated limb − Δsham limb (Table 2). For ECR maximal EMGRMS amplitude, the manipulated limb increased by 9.8 ± 5.7 µV, whereas the sham limb decreased by 4.0 ± 5.3 µV. The resulting difference-in-differences was 13.77 ± 7.55 µV, 95% CI 9.59 to 17.95 µV, t(14) = 7.06, unadjusted *p* < 0.001, and Holm-adjusted *p* = 0.000062. For ECR mean EMGRMS amplitude, the manipulated limb increased by 7.0 ± 4.3 µV, whereas the sham limb decreased by 5.1 ± 4.1 µV. The resulting difference-in-differences was 12.15 ± 5.48 µV, 95% CI 9.11 to 15.18 µV, t(14) = 8.59, unadjusted *p* < 0.001, and Holm-adjusted *p* = 0.000007. No other T2-to-T3 change-score comparison remained statistically significant after Holm–Bonferroni correction. These analyses support a short-term ECR-specific EMG amplitude response after the HVLA procedure, but they should not be interpreted as evidence of improved recovery or performance. These exploratory change-score analyses suggest that the post-intervention ECR difference was not explained solely by the imbalance already present after race 1, but the result should be interpreted cautiously because the study included only 15 participants and was underpowered relative to the a priori sample-size target. Moreover, this result should be interpreted as a short-term difference in ECR EMG amplitude, not as evidence of improved recovery or performance. Because the achieved sample was below the planned participant-level target, non-significant findings for ECU, FCR, FCU, and median frequency outcomes should be interpreted cautiously. These results indicate that no statistically detectable effect was observed in this sample, but they do not exclude smaller or more variable effects that an adequately powered study might detect.

**Figure 2:**
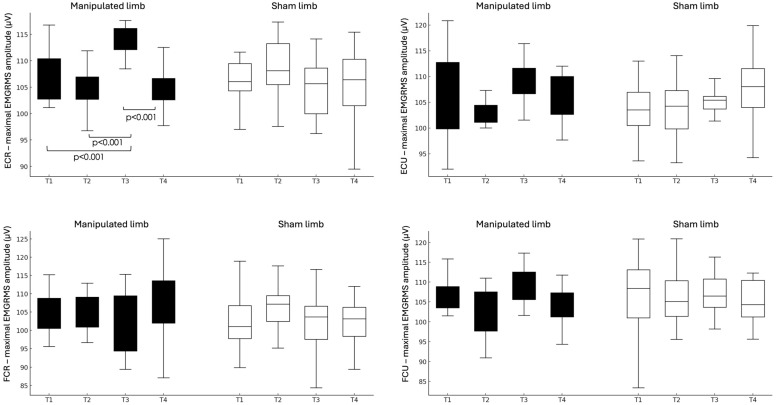
Boxplots of maximal EMG_RMS amplitude (µV) for the extensor carpi radialis (ECR), extensor carpi ulnaris (ECU), flexor carpi radialis (FCR), and flexor carpi ulnaris (FCU) across the four time points: before the race (T1), after race 1 (T2), after the allocated manual procedure (T3), and after race 2 (T4), shown separately for the manipulated limb and sham limb conditions.

For the extensor carpi radialis (ECR), the mixed ANOVA revealed a significant main effect of time, indicating that maximal EMG_RMS amplitude changed across the four measurement points, F(3,42) = 4.40, *p* = 0.009, η^2^ = 0.239. Although the main effect of condition was not significant (*p* = 0.126), there was a significant condition × time interaction for ECR maximal EMG_RMS amplitude, F(3,42) = 8.90, *p* < 0.001, ηp^2^ = 0.389, 95% CI for ηp^2^: 0.139 to 0.555, demonstrating that the temporal pattern of maximal EMG_RMS amplitude differed between the manipulated and sham limbs. In contrast, for the extensor carpi ulnaris (ECU), neither the main effects of condition nor time reached significance (all *p* > 0.05), although a significant cubic interaction contrast emerged (*p* = 0.028, η^2^ = 0.301), suggesting localized non-linear differences between limb conditions over time without a consistent overall interaction. For the flexor carpi radialis (FCR), there were no significant main effects of condition (*p* = 0.136) or time (*p* = 0.327), and the condition × time interaction was also non-significant (*p* = 0.528), indicating stable maximal EMG_RMS amplitude profiles across both limbs over time. Finally, for the flexor carpi ulnaris (FCU), maximal EMG_RMS amplitude showed no significant effects of condition (*p* = 0.486) or time (*p* = 0.242), and the interaction was similarly non-significant (*p* = 0.251); however, strong violations of sphericity were present for this muscle, and Greenhouse–Geisser corrections likewise yielded non-significant outcomes. Figure 2 illustrates the within-condition distributions.

**Figure 3:**
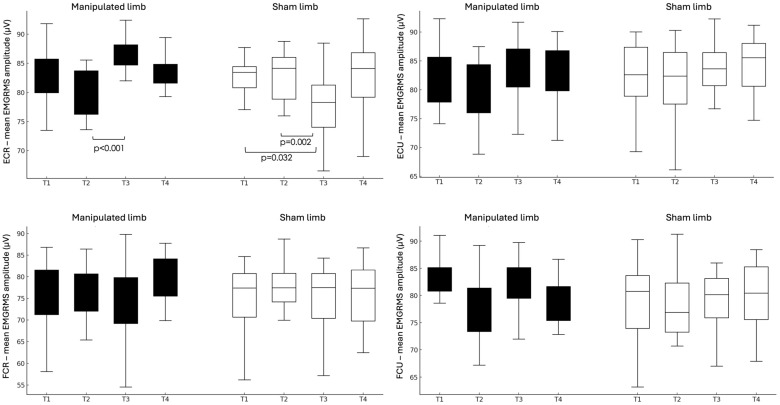
Boxplots of mean EMG_RMS amplitude (µV) for the extensor carpi radialis (ECR), extensor carpi ulnaris (ECU), flexor carpi radialis (FCR), and flexor carpi ulnaris (FCU) across the four time points: before the race (T1), after race 1 (T2), after the allocated manual procedure (T3), and after race 2 (T4), shown separately for the manipulated limb and sham limb conditions.

For mean EMG_RMS amplitude, the mixed ANOVA for the extensor carpi radialis (ECR) showed a significant main effect of condition, F(1,14) = 6.48, *p* = 0.023, η^2^ = 0.316, indicating an overall difference in mean EMG_RMS amplitude between the manipulated and sham limbs. The main effect of time was not significant (*p* = 0.574), but there was a significant condition × time interaction for ECR mean EMG_RMS amplitude, F(3,42) = 8.86, *p* < 0.001, ηp^2^ = 0.388, 95% CI for ηp^2^: 0.138 to 0.554, demonstrating that the temporal pattern of mean EMG_RMS amplitude differed between limb conditions. For the extensor carpi ulnaris (ECU), neither the main effects of condition and time nor their interaction were statistically significant (all *p* ≥ 0.185), although the linear contrast for time approached significance (*p* = 0.092, η^2^ = 0.189), suggesting only a weak tendency for change over time common to both limbs. In the flexor carpi radialis (FCR), no significant main effect of condition was observed, F(1,14) = 0.25, *p* = 0.627, η^2^ = 0.017, and the condition × time interaction was also non-significant, F(3,42) = 0.83, *p* = 0.483, η^2^ = 0.056; the main effect of time did not reach the conventional alpha level either (*p* = 0.095), with a marginal cubic contrast (*p* = 0.052, η^2^ = 0.243) indicating at most a subtle non-linear fluctuation across measurements. Finally, for the flexor carpi ulnaris (FCU), despite marked violations of sphericity, Greenhouse–Geisser-corrected tests confirmed that there were no significant main effects of condition or time and no condition × time interaction for mean EMG_RMS amplitude (all *p* ≥ 0.238, η^2^ ≤ 0.095). Figure 3 illustrates the comparisons.

**Figure 4:**
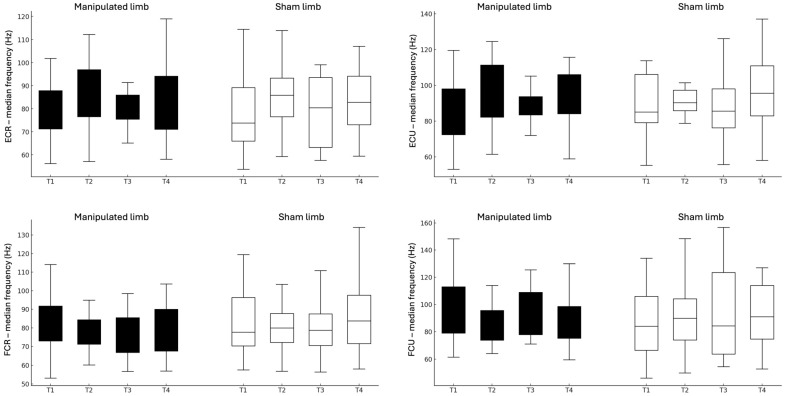
Boxplots of EMG median frequency (Hz) for the extensor carpi radialis (ECR), extensor carpi ulnaris (ECU), flexor carpi radialis (FCR), and flexor carpi ulnaris (FCU) across the four time points: before the race (T1), after race 1 (T2), after the allocated manual procedure (T3), and after race 2 (T4), shown separately for the manipulated limb and sham limb conditions.

For EMG median frequency, the mixed ANOVA for the extensor carpi radialis (ECR) showed no significant main effects of condition or time and no condition × time interaction (all *p* ≥ 0.133, η^2^ ≤ 0.124), indicating that the spectral content of ECR activity was comparable between the manipulated and sham limbs and remained relatively stable across measurements. In contrast, for the extensor carpi ulnaris (ECU), there was a significant main effect of time, F(3,42) = 3.26, *p* = 0.031, η^2^ = 0.189 (Greenhouse–Geisser *p* = 0.041), driven primarily by a significant linear trend (*p* = 0.041, η^2^ = 0.267), suggesting a systematic shift in median frequency over the four time points that was common to both limbs. The condition × time interaction for ECU did not reach the conventional alpha level in the omnibus test (*p* = 0.109), although the quadratic interaction contrast was significant (*p* = 0.020, η^2^ = 0.329), consistent with a subtle, non-linear difference in the temporal pattern between manipulated and sham limbs. For the flexor carpi radialis (FCR), neither the main effect of condition nor the condition × time interaction was significant (*p* = 0.411 and *p* = 0.626, respectively), and the overall time effect was non-significant (*p* = 0.393), though a significant quadratic contrast for time (*p* = 0.035, η^2^ = 0.280) indicated a modest mid-protocol fluctuation in median frequency across both limbs. Finally, for the flexor carpi ulnaris (FCU), there were no significant main effects of condition or time and no condition × time interaction (all *p* ≥ 0.442, η^2^ ≤ 0.056), showing that FCU median frequency remained similar between conditions and across the measurement sequence. Figure 4 illustrates the comparisons.

Exploratory laterality sensitivity analyses were conducted to determine whether the ECR response was influenced by the anatomical side allocated to HVLA manipulation. The manipulated limb was left-sided in seven participants and right-sided in eight participants. For the primary immediate post-procedure interval, defined as the change from post-race 1 to post-intervention (T3–T2), the within-participant between-condition change score was calculated as Δmanipulated limb − Δsham limb. For ECR maximal EMGRMS amplitude, the overall between-condition change was 13.77 ± 7.55 µV, 95% CI 9.59 to 17.95, t(14) = 7.06, *p* < 0.001. When stratified by manipulated-limb side, the change was larger when the manipulated limb was left-sided than when it was right-sided (left: 18.69 ± 8.54 µV; right: 9.47 ± 2.49 µV; left–right difference: 9.22 µV, 95% CI 1.28 to 17.16; Welch t(6.89) = 2.76, *p* = 0.029; Hedges g = 1.43). For ECR mean EMGRMS amplitude, the overall between-condition change was 12.15 ± 5.48 µV, 95% CI 9.11 to 15.18, t(14) = 8.59, *p* < 0.001. This response was also larger in the left-sided than right-sided manipulation sub condition (left: 15.63 ± 5.55 µV; right: 9.10 ± 3.29 µV; left–right difference: 6.54 µV, 95% CI 1.15 to 11.92; Welch t(9.48) = 2.73, *p* = 0.022; Hedges g = 1.37). In Gaussian generalized estimating equation models accounting for repeated measurements within participants, the ECR condition × time effect remained statistically significant after inclusion of manipulated-limb side for both maximal EMGRMS amplitude, Wald χ^2^(3) = 54.70, *p* < 0.001, and mean EMGRMS amplitude, Wald χ^2^(3) = 80.74, *p* < 0.001. However, the condition × time × manipulated-side interaction was also significant for maximal EMGRMS amplitude, Wald χ^2^(3) = 12.37, *p* = 0.006, and mean EMGRMS amplitude, Wald χ^2^(3) = 12.87, *p* = 0.005, indicating that anatomical laterality influenced the magnitude of the immediate ECR amplitude response. These findings suggest that the ECR response was present in both laterality strata but was larger when the manipulated limb was left-sided. Because this was an exploratory sensitivity analysis in a small sample, the laterality finding should be interpreted cautiously and requires confirmation in an adequately powered study with prespecified dominance and side-stratified analyses.

No participant reported sharp pain, paresthesia, dizziness, perceived instability, or unexpected worsening of elbow or forearm symptoms during or immediately after the HVLA or sham procedures. No adverse event required discontinuation of testing or medical referral.

## 4. Discussion

The exploratory present randomized, within-participant sham-controlled trial showed that a single elbow HVLA thrust manipulation, performed after an initial simulated kart race, produced an immediate, muscle-specific change in neuromuscular function of the forearm extensors in trained drivers. Specifically, the primary outcome, ECR mean EMG_RMS amplitude, showed a significant condition × time interaction, with the manipulated limb increasing between post-race 1 and post-intervention while the contralateral sham limb decreased over the same interval. A similar pattern was observed for ECR maximal EMG_RMS amplitude, which was treated as a secondary amplitude outcome. No consistent condition effects were observed for ECU, FCR, or FCU, and EMG median frequency changes over time were small and largely similar in both limbs, indicating that the manipulation did not substantially alter broader forearm EMG responses or classical spectral indices of fatigue. However, because the sample was smaller than planned, the magnitude and precision of this effect require confirmation in adequately powered studies. The comparator was not an untreated contralateral limb, but a sham manual procedure designed to match therapist contact and limb handling while omitting end-range pre-tension and the HVLA thrust. These findings should be interpreted as responses to a specific thrust manipulation protocol, not as evidence for manual therapy or joint mobilization as interchangeable categories. In contrast, no consistent condition effects were observed for the extensor carpi ulnaris (ECU), flexor carpi radialis (FCR), or flexor carpi ulnaris (FCU), and EMG median frequency changes over time were small and largely similar in both limbs, indicating that the manipulation did not substantially alter classical spectral indices of fatigue. These findings should be interpreted as short-term EMG responses to the specific elbow HVLA protocol tested here, not as evidence that spinal manipulation, thoracic mobilization, and peripheral elbow manipulation produce equivalent neurophysiological effects. Because treatment expectation, sham credibility, perceived allocation and prior manual therapy beliefs were not measured, these short-term EMG findings should be interpreted as the overall effect of the delivered HVLA procedure within its clinical context, not as definitive evidence of a thrust-specific biomechanical or neurophysiological mechanism. Additionally, the principal finding should be interpreted narrowly as a transient, muscle-specific modulation of ECR EMG amplitude rather than as evidence of generalized neuromuscular enhancement, improved recovery, increased performance, or injury risk reduction.

An important interpretive issue is the distinction between the karting load-exposure condition and the EMG measurement condition. The simulated races exposed drivers to karting-related demands, including sustained gripping and steering activity, but the EMG outcomes were recorded during static standardized isometric contractions rather than during dynamic driving. Therefore, the observed ECR amplitude response reflects how the forearm muscle responded during a controlled post-load test, not necessarily how it behaved during real-time steering, braking or vibration exposure. Dynamic driving involves changing steering torque, intermittent braking forces, vibration transmission, trunk and shoulder stabilization, visual–motor demands, attentional load and psycho-emotional stress, all of which may alter muscle recruitment patterns relative to a supported isometric laboratory contraction. Thus, the present results should be interpreted as standardized post-load isometric EMG responses rather than as direct evidence of altered neuromuscular control during kart driving.

Regarding maximal EMG_RMS amplitude, the most prominent effect was a significant condition × time interaction for ECR, with higher maximal EMG_RMS amplitude in the sham limb after the first race but a clear reversal after manipulation and a significantly greater positive Δ (T3–T2) in the manipulated limb, while ECU, FCR, and FCU showed no robust between-condition differences across the protocol. This localized enhancement in peak activation is partly consistent with clinical studies in lateral elbow tendinopathy that included Mill’s-type manipulation, although several such protocols combine thrust manipulation with other interventions and therefore do not isolate the independent effect of HVLA manipulation [49]. Systematic reviews of manual procedures for lateral elbow pain suggest possible short-term improvements in pain- and grip-related outcomes, but the underlying interventions are heterogeneous and may include mobilization, manipulation, exercise or multimodal care; consequently, these findings should be regarded as supportive but not definitive evidence for elbow HVLA manipulation specifically [50]. Beyond the elbow, single-session spinal HVLA manipulation has been reported to alter strength-related or corticospinal outcomes in some experimental studies, but extrapolation from spinal manipulation to peripheral elbow manipulation should be made cautiously because the target tissues, afferent input, mechanical dose and motor outputs differ [51]. However, these studies involved spinal manipulation and therefore should not be treated as direct evidence for peripheral elbow HVLA manipulation. The present ECR findings are best interpreted as being compatible with the broader hypothesis that high-velocity articular input can acutely influence neuromuscular output, while recognizing that the specific pathway after elbow manipulation may differ from that after cervical, thoracic or lumbopelvic manipulation. Moreover, surface EMG amplitude is not a direct measure of muscle force, mechanical output, motor-unit behaviour, or sport performance, and muscle force cannot be inferred directly from EMG-derived amplitude without simultaneous force measurement and appropriate modelling [26,39]. Accordingly, the observed increase in ECR amplitude should not be interpreted as enhanced maximal muscle output unless accompanied by corresponding force or performance data.

For mean EMG_RMS amplitude, a similar pattern emerged, with a significant main effect of condition and a robust condition × time interaction for the ECR, whereas ECU, FCR, and FCU showed no significant manipulation-specific changes. Immediately after the first race, the sham limb exhibited higher mean ECR EMG_RMS amplitude than the manipulated limb, but after manipulation the manipulated ECR showed a clear increase and the sham limb a decrease, leading to a substantial between-limb divergence in Δ values. This pattern contrasts with the general tendency for EMG amplitude to increase in parallel with fatigue during sustained or repetitive tasks, as shown in occupational and sports settings, where muscle fatigue is typically characterized by a progressive rise in surface EMG amplitude and concomitant decrements in force output [52]. In motorsport-related models, repetitive braking and power gripping in motorcycle riders have been associated with substantial decrements in maximal grip strength and changes in EMG amplitude of forearm flexors during fatiguing protocols simulating track demands [53]. Likewise, case-study data from track riding indicate that forearm extensors such as the ECR frequently reach a fatigue state during prolonged sessions, with EMG patterns reflecting high activation demands [9]. Against this background, the post-manipulation increase in mean ECR activation, occurring in the absence of a parallel rise in the sham limb, is compatible with several explanations, including a short-term sensorimotor response to the HVLA thrust, nonspecific effects of therapist contact and manual handling, expectation-induced modulation, placebo-related neurophysiological responses, or interaction among these mechanisms. Therefore, the present data do not permit confident attribution of the ECR response to the specific biomechanical or neurophysiological effect of the thrust alone. Experimental work indicates that spinal HVLA manipulation can influence motor-control pathways, including reflex excitability and cortical drive, but whether a peripheral elbow HVLA thrust produces analogous effects in the ECR cannot be determined from the present design [27]. Therefore, the post-manipulation increase in ECR amplitude should be interpreted as a surface EMG observation compatible with short-term sensorimotor facilitation, not as direct evidence that peripheral elbow HVLA manipulation reproduced the central neurophysiological mechanisms reported after spinal manipulation. It cannot be determined from the present data whether this pattern reflects altered voluntary effort, motor-unit recruitment, arousal, expectation, peripheral afferent input, central sensorimotor modulation, or measurement variability. Interpretation of EMG RMS or integrated amplitude as “neuromuscular efficiency” can be misleading in fatiguing contractions, particularly when force output and task performance are not measured simultaneously [54].

The evidence base supporting the present intervention should be viewed hierarchically. Thoracic mobilization studies in lateral epicondylalgia support the possibility that remote non-thrust manual input can influence pain-free grip and sympathetic activity, but they do not test elbow HVLA thrust [33]. Spinal manipulation studies provide mechanistic evidence that thrust procedures may influence cortical or voluntary activation outcomes, but their anatomical target and afferent context differ from those of a peripheral elbow thrust [27,28]. Peripheral elbow manipulation studies are more anatomically relevant but remain limited and have generally emphasized clinical outcomes such as pain and grip strength rather than sport-specific EMG recovery [55]. The present study therefore contributes preliminary peripheral joint data but does not establish whether the observed ECR response was mediated by local mechanoreceptor input, altered nociceptive modulation, spinal reflex pathways, supraspinal motor-control changes, or nonspecific contextual effects.

Expectation, placebo and contextual effects are especially important in manual therapy trials. The therapeutic ritual of positioning, touch, clinician–participant interaction, perceived technical specificity, prior beliefs about manipulation, and expectation of benefit may all influence responses to manual interventions [56]. Although surface EMG is an objective physiological measure, it can still be influenced indirectly by participant effort, arousal, attention, perceived treatment allocation, confidence in the intervention, and immediate motivational or expectancy-related changes during voluntary contraction tasks. The present sham procedure was intended to control for therapist contact, positioning and low-grade movement, but it did not include formal validation of credibility or participant blinding. Accordingly, the observed post-intervention ECR amplitude increase should be interpreted cautiously as a short-term between-condition difference rather than as proof of a specific HVLA thrust mechanism.

In contrast to the amplitude-based variables, EMG median frequency showed only modest time-related changes and no consistent manipulation effect. For ECR, neither condition nor time nor their interaction reached significance, while ECU and FCR demonstrated small but significant or near-significant temporal trends that were largely shared by both limbs, and FCU remained stable throughout. This pattern suggests that the race simulations elicited some degree of neuromuscular fatigue, particularly in ECU, but that the HVLA thrust did not meaningfully modify the spectral correlates of fatigue as captured by median frequency in brief isometric tests. Classical EMG spectroscopy studies have shown that muscle fatigue during sustained or repetitive contractions is typically associated with a progressive shift of the power spectrum towards lower frequencies, reflected in a decline in median frequency [57], and this principle has been confirmed in larger trunk muscles during cyclic lifting tasks [52]. Spectral fatigue methods have also demonstrated acceptable reliability and sensitivity to age- and sex-related changes in back extensor muscles [58]. However, recent work in motorcycle riders has highlighted that median frequency decrements in forearm muscles are not always evident despite substantial reductions in maximal force, particularly when intermittent tasks include relatively generous recovery periods between efforts [53]. In our protocol, the 8 min races were interspersed with seated rest and short isometric test bouts, a configuration that may have allowed partial recovery of conduction velocity and thus attenuated median frequency shifts, while fatigue manifested more prominently as changes in EMG amplitude. A further interpretive issue is the distinction between manual therapy categories. The present study tested a single elbow HVLA thrust manipulation and did not test joint mobilization, mobilization-with-movement, soft-tissue therapy or a multimodal manual therapy package. Mobilization studies in lateral elbow pain are relevant because they show that non-thrust articular input may acutely influence pain-free grip and upper-limb function, but they cannot establish the specific effect of an elbow HVLA thrust. Accordingly, we used mobilization literature only to support biological plausibility and to contextualize the broader manual therapy field, whereas mechanistic interpretation of the present findings was based primarily on thrust-manipulation literature and on the observed within-participant EMG response.

The absence of a consistent manipulation-specific median frequency response further limits interpretation of the amplitude findings as recovery or fatigue reduction. Median frequency is commonly used as a spectral marker of fatigue-related changes in muscle fibre conduction velocity and motor-unit behaviour, but its interpretation is context-dependent and may be influenced by task structure, contraction intensity, recovery intervals, and signal processing choices [57]. Because the present study found no consistent condition effect on median frequency, the results do not provide convincing evidence that HVLA manipulation reduced physiological fatigue. A cautious interpretation of surface EMG is essential. At best, sEMG amplitude provides an estimate of muscle excitation under specific recording, normalization, electrode placement, contraction, and processing conditions; it should not be equated directly with strength, recovery, efficiency, or performance [26]. Changes in EMG amplitude may arise from altered neural drive, motor-unit recruitment or synchronization, muscle fibre conduction properties, electrode–tissue interface changes, contraction strategy, motivation, pain, fatigue, or task execution. Therefore, the ECR amplitude increase observed here should be regarded as a physiological signal requiring confirmation with direct measures of grip force, steering/braking performance, perceived fatigue, pain, and functional outcomes. Additionally, given the reduced sample size, the lack of statistically significant effects in ECU, FCR, FCU and median frequency outcomes should be interpreted conservatively. These null findings suggest that no robust effect was detectable under the present testing conditions, but they cannot rule out smaller, delayed, or more heterogeneous responses in other forearm muscles.

Although the manipulated limb was approximately balanced by anatomical side, laterality appeared to influence the magnitude of the immediate ECR response. The ECR amplitude increase was observed in both left- and right-sided manipulation sub conditions, but the between-condition T2-to-T3 change was larger when the manipulated limb was left-sided. This finding should be interpreted cautiously because the sub condition sizes were small, the analysis was exploratory, and the dataset did not include a separate self-reported dominance variable. Future studies should record limb dominance explicitly, prespecify dominance- and side-stratified analyses, and recruit sufficient participants to test dominance and laterality as potential moderators of the EMG response.

Several limitations should be considered when interpreting these findings. First, although the trial used randomized limb allocation, allocation concealment, assessor blinding, data-analyst blinding, and a standardized sham procedure, the treating physiotherapist could not be blinded and participant blinding was not formally evaluated. Second, the achieved sample size was smaller than planned. The a priori sample-size calculation indicated that 24 participants would be required to detect the hypothesized condition × time interaction with 80% power at α = 0.05, but only 15 drivers were enrolled and analysed, corresponding to approximately 63% of the planned participant level sample. Although each participant contributed two limb observations, the manipulated and sham limbs were paired within the same individual and were not statistically independent; therefore, the 30 limb observations do not compensate for the shortfall in participant level recruitment. Consequently, the study was underpowered, particularly for small-to-moderate effects and for secondary outcomes such as ECU, FCR, FCU, and median frequency variables. This increases the risk of Type II error, meaning that some true but smaller or more variable effects may have gone undetected. Accordingly, non-significant findings for muscles other than ECR and for spectral fatigue indices should be interpreted as inconclusive rather than as definitive evidence of no effect.

The comparator should also be interpreted carefully. This was a within-participant sham-controlled limb allocation study, not a parallel-group randomized trial and not a no-treatment contralateral-control design. The sham procedure controlled for therapist contact, limb handling, and approximate procedure duration, but it may not have fully controlled for sensory differences associated with the HVLA thrust. Moreover, the intervention consisted of a single manipulation session, and outcomes were followed only over the course of one experimental day. Thus, no inferences can be made about longer-term adaptation, cumulative effects of repeated manipulations, or clinical outcomes such as pain or tendinopathy incidence. Additionally, there was a deliberate but important gap between the dynamic loading condition and the EMG measurement condition. Although the simulated races were used to impose karting-specific forearm load, EMG outcomes were collected during standardized isometric laboratory tests rather than during actual dynamic driving. The laboratory tests used fixed joint positions, supported forearm posture and standardized resistance, whereas kart driving involves dynamic steering, variable steering and braking forces, vibration, whole-body stabilization, environmental demands, attentional load, and psycho-emotional stress. Consequently, it remains uncertain how closely the observed isometric ECR response reflects neuromuscular behaviour during real driving or competitive racing. The findings should therefore be interpreted as controlled post-load isometric EMG responses and not as direct evidence of EMG behaviour during real-time steering, braking, vibration exposure, race-like psychological stress, or competitive karting. Additionally, while the within-participant design and bilateral EMG recordings control for many inter-individual confounders, the treating physiotherapist was necessarily unblinded, and subtle expectation or interaction effects cannot be entirely excluded. The sham limb received a standardized sham manual contact procedure designed to match therapist presence, touch, limb positioning, handling, and approximate duration; however, sham credibility and participant perception of allocation were not formally assessed, so the comparator should be interpreted as a procedural sham control rather than as a fully validated placebo. A further limitation concerns mechanistic inference. The rationale for this trial partly drew on spinal manipulation and thoracic mobilization literature, but those procedures differ from peripheral elbow HVLA manipulation in anatomical target, mechanical dose, afferent input and expected neurophysiological response. The present study did not include neurophysiological measures such as transcranial magnetic stimulation, H-reflex testing, autonomic markers, pain–pressure thresholds, mechanoreceptor assessment or motor unit decomposition; therefore, the mechanism underlying the observed ECR amplitude change remains uncertain. Future studies should directly compare spinal manipulation, peripheral elbow HVLA manipulation, non-thrust elbow mobilization, and sham manual contact while including mechanistic outcomes capable of separating local, spinal, supraspinal, and contextual effects. Future studies should compare HVLA manipulation, non-thrust mobilization, sham manual contact, and no-treatment conditions in separate arms to determine whether the observed EMG response is specific to the thrust component, to manual contact, or to nonspecific expectation and contextual effects. Another important limitation is that EMG amplitude was used as a physiological outcome rather than as a direct marker of performance or recovery.

The study also cannot address the management of post-traumatic elbow stiffness or symptomatic lateral elbow tendinopathy. Although these conditions are clinically relevant in motorsport and elbow rehabilitation, participants with recent upper-extremity injury, pain, surgery or clinically relevant pathology were excluded. Moreover, the study did not compare HVLA manipulation with conservative stiffness interventions such as splinting, exercise or non-thrust mobilization, nor did it evaluate surgical indications or postoperative outcomes. Future studies should separately investigate clinical populations with lateral elbow tendinopathy, post-traumatic elbow stiffness and healthy sport-related forearm fatigue, rather than combining these entities within a single therapeutic rationale. Moreover, future manual therapy trials in karting drivers should include validated measures of expectation, credibility and blinding success.

From a practical perspective, the present results suggest that the specific lateral elbow HVLA protocol tested here may acutely increase ECR activation after an initial simulated race, but they do not show that manual therapy in general, joint mobilization, or multimodal elbow treatment would produce the same response. They show only that the tested HVLA procedure was associated with transient ECR EMG amplitude modulation during a standardized isometric test performed after simulated karting load. They do not establish that the intervention modifies forearm activation during dynamic steering, braking, vibration exposure or competitive driving. However, the study does not demonstrate improved recovery, enhanced neuromuscular efficiency, increased grip strength, better steering or braking performance, reduced fatigue, pain reduction, or injury prevention. In practice, any use of elbow HVLA manipulation in karting drivers should remain clinically individualized, performed only by appropriately trained clinicians, and considered preliminary and unproven with respect to performance enhancement, because the present study measured short-term EMG responses rather than race performance, pain reduction or injury prevention. Moreover, results should not be interpreted as support for HVLA manipulation in drivers with recent trauma, fracture, postoperative restriction, post-traumatic stiffness, instability, active elbow pain or suspected tendinopathy without appropriate clinical assessment. In such cases, management should follow condition-specific clinical reasoning, which may include rehabilitation, load management, exercise, splinting, non-thrust mobilization, imaging or surgical referral, depending on the diagnosis. However, given the absence of effects on other forearm muscles and on spectral fatigue indices, such techniques should be viewed as an adjunct rather than a substitute for a comprehensive approach that address strength, endurance and load management of the upper limb, as recommended in broader motorsport and lateral elbow rehabilitation literature. Future studies should therefore test whether the observed short-term ECR EMG_RMS response is reproducible and whether it has any relationship to separately measured force, range-of-motion, kinematic, fatigue-specific, clinical, or performance outcomes.

## 5. Conclusions

A single HVLA manipulation of the elbow produced a short-term, muscle-specific increase in the primary outcome, ECR mean EMG_RMS amplitude, after simulated karting load. A secondary ECR maximal EMG_RMS amplitude response showed a similar direction of effect, whereas exploratory outcomes from ECU, FCR, FCU, and EMG median frequency did not provide evidence of a consistent broader forearm response. These findings should be interpreted strictly as short-term electrophysiological responses measured during standardized isometric testing. They do not show changes in handgrip force, elbow range of motion, upper-limb kinematics, neuromuscular efficiency, fatigue resistance, karting performance, pain, clinical benefit or injury prevention, because those outcomes were not analysed in the present report. Because evidence from spinal manipulation and thoracic mobilization cannot be assumed to generalize directly to peripheral elbow manipulation, the present results should be interpreted as preliminary elbow-specific observations rather than confirmation of a shared neurophysiological mechanism across manual therapy techniques. The results should not be generalized to post-traumatic elbow stiffness, recent traumatic elbow injury, postoperative restriction or symptomatic lateral elbow tendinopathy, because these conditions have distinct mechanisms, contraindications and management pathways. Moreover, because EMG was recorded during static isometric laboratory tests rather than during dynamic steering, braking, vibration exposure, or competitive racing, the results cannot be generalized to real-time forearm muscle behaviour during kart driving. The present results should be interpreted cautiously and cannot be generalized to sustained training effects or clinical endpoints such as pain or injury risk. Within these limitations, the tested elbow HVLA manipulation protocol may be considered a preliminary physiological intervention requiring further validation for short-term modulation of forearm extensor activation in kart drivers, but its practical relevance remains to be confirmed and should not be generalized to joint mobilization or manual therapy more broadly.

## Figures and Tables

**Table 1:** Mean and standard deviations for all muscle sites and outcomes across the four assessment time points for the manipulated and sham limb conditions.

Muscle and Outcome	Condition	Baseline	After Race 1	After Manipulation	After Race 2
ECR—maximal EMG_RMS amplitude (µV)	Manipulated limb	106.2 ± 6.3	104.5 ± 3.7	114.3 ± 4.9	105.1 ± 4.1
	Sham limb	105.7 ± 5.3	108.2 ± 6.2	104.2 ± 7.5	105.0 ± 7.4
	*p* (raw/Holm)	0.802/1.000	0.008/0.354	<0.001/0.010 *	0.985/1.000
ECR—mean EMG_RMS amplitude (µV)	Manipulated limb	82.8 ± 5.5	79.8 ± 4.3	86.9 ± 3.4	83.7 ± 3.9
	Sham limb	82.5 ± 4.0	82.9 ± 4.5	77.7 ± 6.1	82.4 ± 7.8
	*p* (raw/Holm)	0.851/1.000	0.005/0.243	<0.001/0.004 *	0.521/1.000
ECR—median frequency (Hz)	Manipulated limb	79.9 ± 13.5	86.6 ± 14.5	80.9 ± 9.8	78.6 ± 28.3
	Sham limb	76.5 ± 16.6	85.0 ± 17.0	79.3 ± 15.4	84.0 ± 15.2
	*p* (raw/Holm)	0.571/1.000	0.746/1.000	0.639/1.000	0.543/1.000
ECU—maximal EMG_RMS amplitude (µV)	Manipulated limb	104.5 ± 11.0	102.5 ± 3.3	109.1 ± 7.5	105.5 ± 6.1
	Sham limb	103.8 ± 6.6	103.9 ± 5.6	104.5 ± 5.0	107.9 ± 6.3
	*p* (raw/Holm)	0.822/1.000	0.311/1.000	0.063/1.000	0.353/1.000
ECU—mean EMG_RMS amplitude (µV)	Manipulated limb	80.6 ± 8.6	79.4 ± 7.1	83.5 ± 5.2	82.1 ± 7.1
	Sham limb	82.3 ± 5.9	81.7 ± 6.8	83.2 ± 5.7	84.3 ± 4.7
	*p* (raw/Holm)	0.481/1.000	0.317/1.000	0.835/1.000	0.371/1.000
ECU—median frequency (Hz)	Manipulated limb	85.8 ± 20.6	96.7 ± 20.7	88.2 ± 13.9	92.9 ± 18.2
	Sham limb	89.0 ± 18.7	89.5 ± 14.4	88.6 ± 20.3	96.8 ± 22.5
	*p* (raw/Holm)	0.355/1.000	0.089/1.000	0.931/1.000	0.447/1.000
FCR—maximal EMG_RMS amplitude (µV)	Manipulated limb	104.9 ± 8.8	105.0 ± 5.3	102.0 ± 9.2	108.1 ± 10.5
	Sham limb	102.6 ± 11.2	105.4 ± 7.2	100.7 ± 11.6	101.9 ± 6.7
	*p* (raw/Holm)	0.561/1.000	0.858/1.000	0.610/1.000	0.104/1.000
FCR—mean EMG_RMS amplitude (µV)	Manipulated limb	76.1 ± 9.0	75.7 ± 7.3	72.6 ± 10.9	79.6 ± 5.4
	Sham limb	74.7 ± 9.0	76.4 ± 7.8	73.9 ± 9.7	75.7 ± 7.2
	*p* (raw/Holm)	0.660/1.000	0.825/1.000	0.677/1.000	0.062/1.000
FCR—median frequency (Hz)	Manipulated limb	82.0 ± 15.3	80.1 ± 14.6	76.4 ± 13.0	80.8 ± 17.8
	Sham limb	83.0 ± 18.9	79.4 ± 13.9	80.1 ± 15.3	85.4 ± 21.1
	*p* (raw/Holm)	0.782/1.000	0.786/1.000	0.256/1.000	0.371/1.000
FCU—maximal EMG_RMS amplitude (µV)	Manipulated limb	106.7 ± 4.1	102.7 ± 6.8	130.9 ± 87.3	102.3 ± 7.8
	Sham limb	105.9 ± 11.7	106.5 ± 6.9	107.0 ± 5.1	105.0 ± 5.7
	*p* (raw/Holm)	0.815/1.000	0.119/1.000	0.303/1.000	0.313/1.000
FCU—mean EMG_RMS amplitude (µV)	Manipulated limb	82.7 ± 3.9	77.8 ± 6.3	99.6 ± 70.0	77.6 ± 6.4
	Sham limb	78.4 ± 8.9	78.4 ± 6.6	78.8 ± 5.7	80.1 ± 6.0
	*p* (raw/Holm)	0.096/1.000	0.783/1.000	0.262/1.000	0.303/1.000
FCU—median frequency (Hz)	Manipulated limb	96.1 ± 24.7	89.0 ± 21.0	92.2 ± 18.8	88.2 ± 17.4
	Sham limb	91.8 ± 35.7	89.9 ± 25.2	95.9 ± 34.2	96.2 ± 33.1
	*p* (raw/Holm)	0.704/1.000	0.876/1.000	0.685/1.000	0.276/1.000

ECR: extensor carpi radialis; ECU: extensor carpi ulnaris; FCR: flexor carpi radialis; FCU: flexor carpi ulnaris; EMG_RMS: root mean square electromyographic amplitude; EMG median frequency is expressed in Hz. Limb conditions were classified as manipulated limb and sham limb. EMG_RMS amplitude values represent surface-recorded muscle excitation during standardized isometric testing and are expressed in µV; they should not be interpreted as mechanical force. *: significantly different for *p* < 0.05.

**Table 2:** Primary and secondary ECR condition × time estimates with exploratory T2-to-T3 difference-in-differences contrasts.

Outcome	Omnibus Condition × Time Interaction	ηp^2^ (95% CI)	Immediate T2–T3 Difference-in-Differences, Mean ± SD (µV)	95% CI for Difference-in-Differences (µV)	Paired Test for Difference-in-Differences	Holm-Adjusted *p*
Primary outcome: ECR mean EMG_RMS amplitude (µV)	F(3,42) = 8.86; *p* < 0.001	0.388 (0.138 to 0.554)	12.15 ± 5.48	9.11 to 15.18	t(14) = 8.59; unadjusted *p* < 0.001	0.000007
Secondary outcome: ECR maximal EMG_RMS amplitude (µV)	F(3,42) = 8.90; *p* < 0.001	0.389 (0.139 to 0.555)	13.77 ± 7.55	9.59 to 17.95	t(14) = 7.06; unadjusted *p* < 0.001	0.000062

ECR: extensor carpi radialis; EMG_RMS: root mean square electromyographic amplitude; ηp^2^: partial eta-squared; CI: confidence interval; T2: after race 1; T3: post-intervention. The immediate T2–T3 difference-in-differences was calculated as Δmanipulated limb − Δsham limb, where Δ = T3 − T2. Values for the difference-in-differences are mean ± SD of the paired within-participant contrast. Holm-adjusted *p* values refer to the exploratory family of 12 T2-to-T3 change-score comparisons (4 muscles × 3 EMG outcomes).

## Data Availability

The data can be provided upon a reasonable request to the corresponding author.
